# Nuclear Imaging in Renal Cell Carcinoma: Current Evidence and Clinical Applications

**DOI:** 10.3390/cancers18020195

**Published:** 2026-01-07

**Authors:** Abdullah Al-Khanaty, Shane Qin, Carlos Delgado, David Hennes, Eoin Dinneen, David Chen, Lewis Au, Renu S. Eapen, Damien Bolton, Declan G. Murphy, Nathan Lawrentschuk, Gregory Jack, Daniel Moon, Michael S. Hofman, Marlon L. Perera

**Affiliations:** 1 Division of Cancer Surgery, Peter MacCallum Cancer Centre, 305 Grattan Street, Melbourne 3000, Australiamarlon.perera@unimelb.edu.au (M.L.P.); 2 Department of Surgery, University of Melbourne, Austin Health, Melbourne 3084, Australia; 3 Hudson Institute of Medical Research, Monash University, Clayton 3800, Australia; 4 Sir Peter MacCallum Department of Oncology, University of Melbourne, Melbourne 8006, Australia; 5 Department of Medical Oncology, Peter MacCallum Cancer Centre, Melbourne 3000, Australia; 6 Department of Surgery, University of Melbourne, Royal Melbourne Hospital, Melbourne 3000, Australia; 7 EJ Whitten Prostate Cancer Research Centre at Epworth, Melbourne 3001, Australia; 8 Department of Molecular Imaging and Therapeutic Nuclear Medicine, Cancer Imaging, Peter MacCallum Cancer Centre, Melbourne 3000, Australia; 9 Prostate Cancer Theranostics and Imaging Centre of Excellence (ProsTIC), Peter MacCallum Cancer Centre, Melbourne 3000, Australia

**Keywords:** renal cell carcinoma, PET/CT, [^18^F]FDG, PSMA-targeted imaging, carbonic anhydrase IX, fibroblast activation protein inhibitor, nuclear imaging, clear-cell renal cell carcinoma

## Abstract

**Introduction:** Radiotracer-based nuclear imaging, including positron emission tomography (PET) and single-photon emission computed tomography (SPECT), can complement conventional cross-sectional imaging in renal cell carcinoma (RCC) by providing biological characterisation of tumour metabolism, angiogenesis, hypoxia, and the tumour microenvironment. While computed tomography (CT) and magnetic resonance imaging (MRI) remain the diagnostic standard, accumulating evidence suggests that selected nuclear imaging techniques may offer incremental value in specific clinical scenarios. **Methods:** A narrative literature review was performed using PubMed, Embase, and Web of Science to identify preclinical, retrospective, and prospective studies evaluating PET and SPECT radiotracers in localised and metastatic RCC. Priority was given to meta-analyses, multicentre prospective trials, and studies with histopathological correlation. **Results:** [^18^F]fluorodeoxyglucose (FDG) PET/CT demonstrates limited sensitivity for primary renal tumours (pooled sensitivity of approximately 60%) but performs substantially better in metastatic and recurrent disease (pooled sensitivity and specificity of approximately 85–90%), where uptake correlates with tumour grade, progression-free survival, and overall survival. [^99m^Tc]sestamibi SPECT/CT differentiates oncocytoma and hybrid oncocytic/chromophobe tumours from malignant RCC with pooled sensitivity and specificity of around 85–90%, supporting its role in evaluating indeterminate renal masses rather than staging. Prostate-specific membrane antigen (PSMA) PET/CT shows high detection rates in clear-cell RCC, particularly in metastatic disease, with reported sensitivities of approximately 85–90% and management changes in up to 40–50% of selected cohorts. Carbonic anhydrase IX (CAIX)-targeted PET/CT enables the biologically specific visualisation of clear-cell RCC, achieving sensitivities and specificities in the range of 85–90% in prospective phase II and III trials for primary tumour characterisation. Fibroblast activation protein inhibitor (FAPI) PET/CT demonstrates high tumour-to-background uptake in early RCC studies, but evidence remains preliminary, with small cohorts and recognised non-specific uptake in benign inflammatory and fibrotic conditions. **Conclusions:** Radiotracer-based nuclear imaging provides complementary, biology-driven insights in RCC that extend beyond anatomical assessment. While most modalities remain adjunctive or investigational and are not recommended for routine use, selective application in carefully chosen clinical scenarios may enhance tumour characterisation, prognostication, and personalised treatment planning.

## Simple Summary

Standard imaging for kidney cancer mainly shows the size and location of tumours but provides limited information about tumour biology. Radiotracer-based nuclear imaging uses specialised tracers to visualise cancer metabolism, blood supply, and the tumour microenvironment, offering additional biological insight. This review summarises current evidence of the nuclear imaging used in renal cell carcinoma, including [^18^F]FDG PET/CT, PSMA PET/CT, [^99m^Tc]-sestamibi SPECT/CT, CAIX PET/CT, and FAPI PET/CT. [^18^F]FDG PET is useful for staging and prognosis in advanced disease. PSMA PET may improve the detection of metastatic clear-cell renal cancer and can influence treatment decisions. Sestamibi imaging may help distinguish benign oncocytic tumours from malignant renal cancers, potentially avoiding unnecessary surgery. CAIX and FAPI tracers represent emerging tools that may support more precise diagnosis and targeted therapies. Together, these nuclear imaging techniques may enhance personalised care for patients with kidney cancer.

## 1. Introduction

Renal cell carcinoma (RCC) represents a global health burden, with approximately 403,000 new diagnoses annually, accounting for around 2–3% of all cancers worldwide [1]. The disease shows a male predominance (relative risk ≈ 1.7) and the highest incidence in North America, Western Europe, and Oceania, partly due to the widespread use of cross-sectional abdominal imaging [1]. Although RCC incidence has risen gradually over recent decades, mortality has modestly declined, driven by earlier detection and stage migration, advances in systemic therapies, and an increasing contribution from the overdiagnosis of small, indolent renal tumours detected incidentally on cross-sectional imaging.

RCC is a biologically heterogeneous disease encompassing several histologic subtypes with distinct molecular and radiologic characteristics. Clear-cell RCC (ccRCC), which accounts for 70–80% of cases, is defined by biallelic von Hippel–Lindau (VHL) gene inactivation and subsequent hypoxia-inducible factor (HIF) pathway activation, driving angiogenesis, altered metabolism, and the expression of downstream targets such as carbonic anhydrase IX (CAIX) [2]. Papillary and chromophobe RCCs, in contrast, are driven by different molecular pathways including MET activation and mitochondrial dysfunction, which influence both their imaging phenotypes and therapeutic responses [3].

There are currently no validated blood-based biomarkers for RCC, and diagnosis is often made incidentally on imaging performed for unrelated reasons [2]. Most incidentally detected renal tumours are small (<4 cm) and correspond to stage T1 disease, which is largely curable with surgery. However, up to 20% of these lesions are benign, including oncocytomas and hybrid oncocytic/chromophobe tumours (HOCTs) [4].

Conventional contrast-enhanced computed tomography (CT) and magnetic resonance imaging (MRI) remain the diagnostic mainstay for evaluating renal masses, offering excellent anatomic resolution for staging and surgical planning. Nonetheless, these modalities have important limitations, as they cannot reliably distinguish benign from malignant lesions, define histologic subtypes, or accurately assess treatment response, particularly following targeted or immune-based therapies that induce necrosis or atypical enhancement patterns [5]. Although percutaneous renal mass biopsy can provide a tissue diagnosis, its role remains limited due to non-diagnostic rates of up to 22%, sampling error related to tumour heterogeneity, and challenging tumour locations [6]. As a result, many indeterminate renal masses are surgically removed, contributing to the overtreatment of benign or indolent disease [7].

Nuclear imaging techniques, including positron emission tomography (PET) and single-photon emission computed tomography (SPECT), can complement CT and MRI by providing functional and molecular information beyond anatomic imaging alone. By targeting metabolic, angiogenic, hypoxia-related, or stromal pathways, these modalities may assist with tumour characterisation, biopsy risk stratification, and the assessment of treatment response in selected settings [8].

Despite growing interest, international guidelines do not recommend routine PET or SPECT imaging for the diagnosis, staging, or surveillance of RCC. The European Association of Urology (EAU) (2025) [9] acknowledges emerging evidence supporting PSMA PET/CT, [^99m^Tc]sestamibi SPECT/CT, and ^89^Zr-DFO-girentuximab PET/CT for subtype discrimination and biological characterisation, but concludes that external validation and outcome data remain insufficient for guideline endorsement. The American Urological Association (AUA) (2021) [10] similarly advises against routine PET imaging, noting a limited role for problem-solving in suspected recurrence or metastatic disease when conventional imaging is inconclusive. The European Society for Medical Oncology (ESMO) (2024) [11] does not include PET or SPECT imaging in recommended diagnostic or staging pathways. The National Comprehensive Cancer Network (NCCN) (2025) [12] likewise does not endorse routine PET imaging, but acknowledges the selective use of [^18^F]FDG PET/CT in equivocal, aggressive, or recurrent disease.

Accordingly, the nuclear imaging modalities discussed in this review should be interpreted as adjunctive or investigational tools rather than replacements for standard cross-sectional imaging pathways. This review summarises the role of radiotracer-based nuclear imaging in RCC, focusing on [^18^F]FDG, PSMA, CAIX, [^99m^Tc]-sestamibi, and FAPI, and highlights their diagnostic and prognostic implications within the context of contemporary evidence and current guideline recommendations.

## 2. Methods

This narrative review synthesises contemporary evidence on the clinical application of PET and SPECT in RCC. A targeted literature search was undertaken across PubMed, Embase, and Web of Science from database inception to October 2025. Search terms combined disease-specific and imaging-related keywords, including “renal cell carcinoma” or “RCC” together with “PET,” “SPECT,” “FDG,” “PSMA,” “CAIX,” “fibroblast activation protein inhibitor (FAPI),” and “sestamibi,” with syntax adapted for individual databases. Reference lists of key publications were manually screened to identify additional relevant studies.

Eligible studies included prospective and retrospective clinical series, multicentre trials, meta-analyses, and translational studies evaluating PET or SPECT imaging in adults with RCC or indeterminate renal masses. Case reports, very small series (fewer than five patients), non-clinical studies, and non-English publications were excluded. Where overlapping cohorts were identified, the most comprehensive or most recent report was prioritised. Given the heterogeneity of imaging protocols, tracers, patient populations, and clinical endpoints, the findings were integrated qualitatively rather than pooled quantitatively.

Study selection was performed through title and abstract screening followed by full-text review. Evidence was organised by radiotracer type and clinical application, including the characterisation of indeterminate renal masses, staging of locoregional or metastatic disease, prognostic assessment, therapy response monitoring, and emerging theranostic approaches. Greater interpretative weight was placed on prospective studies, multicentre cohorts, and investigations incorporating histopathological correlation or predefined clinical endpoints, while smaller retrospective series were contextualised accordingly.

To contextualise clinical adoption, contemporary guideline documents from the EAU (2025) [9], AUA (2021) [10], ESMO (2024) [13], and NCCN (2025) [12] guidelines were manually reviewed. Statements regarding the role of PET and SPECT imaging were interpreted with reference to specific guideline recommendations across diagnostic, staging, surveillance, and treatment–response settings.

Formal risk-of-bias tools were not applied, as this work was conceived as a narrative rather than systematic review. Nonetheless, the interpretation of diagnostic accuracy and management–impact data explicitly considered recognised sources of bias and applicability, including patient selection, reference standards, lesion-based versus patient-based analyses, imaging acquisition and interpretation, and cohort size. This approach aimed to provide a balanced synthesis of the current evidence while acknowledging the limitations and heterogeneity of the existing literature.

## 3. [^99m^Tc]Tc-Sestamibi SPECT/CT Imaging in Renal Cell Carcinoma

### 3.1. Biological and Molecular Basis

[^99m^Tc]Tc-sestamibi is a lipophilic cationic radiopharmaceutical long established in cardiac, parathyroid, and breast imaging. Cellular uptake is governed by mitochondrial membrane potential and intracellular retention is influenced by multidrug-resistant (MDR) efflux pump expression, particularly P-glycoprotein [4,5]. In renal neoplasia, these biological determinants generate distinctive uptake patterns: oncocytomas and hybrid oncocytic/chromophobe tumours (HOCTs) are typically sestamibi-avid due to high mitochondrial density, whereas many clear-cell and papillary RCCs demonstrate reduced or absent uptake associated with increased MDR activity [14]. This metabolic divergence underpins the use of sestamibi SPECT/CT as a functional imaging technique for the risk stratification of indeterminate renal masses, with the aim of improving confidence in benign or indolent pathology and potentially reducing unnecessary surgical intervention. In contrast to CAIX- or PSMA-targeted PET tracers, which have applications in locoregional and metastatic assessment, sestamibi SPECT/CT remains largely confined to primary renal mass characterisation rather than staging [14].

Early mechanistic work by Rowe et al. established the biological basis for sestamibi uptake in renal tumours, demonstrating intense tracer accumulation in oncocytomas and minimal uptake in clear-cell RCC in initial translational series [14]. These observations were subsequently validated in a prospective single-centre clinical study by Gorin et al. [15] that evaluated [^99m^Tc]-sestamibi SPECT/CT in 50 patients with cT1 solid renal masses undergoing surgical resection. Using blinded dual-reader interpretation and surgical histopathology as the reference standard, [^99m^Tc]-sestamibi SPECT/CT correctly identified five of six oncocytomas and both HOCTs, yielding a sensitivity of 87.5% (95% CI 47.4–99.7%). Only two lesions were falsely positive, resulting in a specificity of 95.2% (95% CI 83.8–99.4%). Although limited by small numbers of oncocytic tumours and a selected surgical cohort, this study provided early prospective evidence that [^99m^Tc]-sestamibi SPECT/CT can non-invasively discriminate oncocytic renal neoplasms from RCC in appropriately selected patients. Correlative analyses confirmed that [^99m^Tc]-sestamibi uptake reflects tumour bioenergetic phenotype, correlating positively with mitochondrial abundance and inversely with MDR-1 expression rather than tumour size or grade, providing a biologically plausible basis for its role in the non-invasive characterisation of renal masses.

### 3.2. [^99m^Tc]Tc-Sestamibi SPECT/CT’s Diagnostic Role in Indeterminate Renal Masses

Most studies evaluating [^99m^Tc]-sestamibi SPECT/CT have been conducted in selected cohorts of patients with indeterminate or diagnostically challenging renal masses rather than unselected all-comer RCC populations (Table 1). In these studies, [^99m^Tc]-sestamibi imaging was typically requested following inconclusive cross-sectional imaging or when oncocytic pathology was suspected, reflecting its intended role as a problem-solving adjunct rather than a screening or staging modality. As a result, reported sensitivity and specificity estimates should be interpreted in the context of cohort enrichment and pre-test probability, particularly given the over-representation of oncocytoma, HOCTs, and chromophobe RCC relative to routine clinical practice.

Early single-centre prospective studies have reported encouraging diagnostic performance for [^99m^Tc]-sestamibi SPECT/CT in the evaluation of indeterminate renal masses. In a prospective cohort of 29 patients encompassing 31 renal masses, Sistani et al. [7] demonstrated sestamibi uptake in all oncocytic lesions (seven oncocytomas and one HOCT), while the majority of RCC subtypes—including clear-cell and papillary RCC—were sestamibi-negative. Using visual and quantitative tumour-to-parenchyma uptake assessment, this translated to a reported sensitivity of 100% and specificity of 96% for distinguishing benign oncocytic lesions from RCC in this enriched referral population. One chromophobe RCC exhibited low-grade tracer uptake, highlighting the known overlap in mitochondrial biology between chromophobe and oncocytic tumours. Similarly, Parihar et al. [16] assessed solid renal masses undergoing additional diagnostic work-up and found [^99m^Tc]-sestamibi SPECT/CT to outperform contrast-enhanced CT, particularly when quantitative tumour-to-renal parenchyma uptake ratios were applied. Importantly, both studies were explicitly performed in enriched indeterminate cohorts, limiting the generalisability of point estimates to broader RCC populations. In a prospective single-centre cohort of 74 patients with small renal masses, Viswambaram et al. [17] evaluated [^99m^Tc]-sestamibi SPECT/CT against histopathology and reported a sensitivity of 89% (95% CI 77–95%) and specificity of 73% (95% CI 45–91%) for malignancy detection. A visually “cold” scan demonstrated a high PPV of 92%, whereas the NPV of a “hot” scan was lower at 65%, indicating that sestamibi negativity reliably supports malignancy, but sestamibi avidity does not exclude malignancy when used alone.

Quantitative analysis identified an optimal tumour-to-kidney uptake ratio of 0.41 (AUC 0.883, 95% CI 0.794–0.971), though this did not improve diagnostic accuracy beyond visual interpretation. These findings highlight [^99m^Tc]-sestamibi SPECT/CT as a useful adjunctive tool rather than a standalone discriminator in clinical decision-making. In a real-world, single-centre implementation study, Schober et al. [18] evaluated 71 patients (88 masses) undergoing [^99m^Tc]-sestamibi SPECT/CT, with pathology available for 52 lesions. Among lesions interpreted as “cold,” 20% were ultimately oncocytomas or HOCTs on histopathology, corresponding to a miss rate that limits the use of sestamibi negativity as a definitive rule-in for malignancy. When biopsy-only cases were excluded, the negative predictive value of a “cold” scan for malignancy was 87.5%. Formal confidence intervals were not reported, reflecting the pragmatic, real-world design. These findings underscore the fact that NPV and miss rate are more clinically informative than sensitivity alone when sestamibi imaging is used to guide surveillance or biopsy avoidance.

Systematic reviews and meta-analyses reinforce these findings while clarifying their limitations. In the first PRISMA-DTA meta-analysis, Wilson et al. [19] pooled four diagnostic accuracy studies encompassing 117 renal lesions and reported a pooled sensitivity of 92% (95% CI 72–98%) and specificity of 88% (95% CI 79–94%) for differentiating oncocytoma from other renal lesions using [^99m^Tc]-sestamibi SPECT/CT. However, specificity fell to 67% in pooled analyses comparing oncocytoma with chromophobe RCC, reflecting biological overlap in mitochondrial-rich tumours. More recently, Basile et al. [4] conducted an updated PRISMA-registered meta-analysis of eight prospective and retrospective studies encompassing 501 indeterminate renal masses. In pooled analyses, [^99m^Tc]-sestamibi SPECT/CT demonstrated a sensitivity of 89% (95% CI 70–97%) and specificity of 89% (95% CI 86–92%) for differentiating oncocytoma/HOCT from other renal lesions. Importantly, the pooled NPV was high at 98% (95% CI 92–100%), whereas the PPV was lower at 63% (95% CI 46–76%), reflecting a greater ability to exclude malignancy than to definitively confirm benign histology. Diagnostic performance was strongest when oncocytoma/HOCT was compared against clear-cell and papillary RCC (pooled specificity 98%, 95% CI 91–100%), but substantially weaker for chromophobe RCC (pooled specificity 41%, 95% CI 23–62%), underscoring a key biological and clinical limitation of sestamibi imaging in this mitochondrial-rich subgroup.

### 3.3. Quantitative Assessment and Interpretation

The quantitative interpretation of [^99m^Tc]-sestamibi SPECT/CT commonly employs tumour-to-renal parenchyma uptake ratios, with reported thresholds ranging from approximately 0.46 to 0.65 across studies [14,16]. Lesions exceeding these thresholds are more likely to represent oncocytic or indolent histology, while lower uptake raises suspicion for RCC. Quantitative assessment may be particularly useful for lesions with equivocal visual uptake, although most studies demonstrate high interobserver agreement using qualitative interpretation alone. The incorporation of morphological CT features such as cystic change or necrosis may further refine diagnostic confidence. While spatial resolution is inferior to PET/CT, [^99m^Tc]-sestamibi SPECT/CT has demonstrated consistent performance in small renal masses, with reported sensitivities approaching 85–90% [10].

### 3.4. Clinical Applications and Impact

Clinically, [^99m^Tc]-sestamibi SPECT/CT is best positioned as a risk-stratification tool for indeterminate renal masses prior to biopsy or surgical intervention. A sestamibi-avid (“hot”) lesion increases confidence in benign or indolent pathology and may support active surveillance or biopsy avoidance in appropriately selected patients. However, given the modest negative predictive value reported in prospective and real-world series, sestamibi negativity should not be interpreted as definitive evidence of malignancy in isolation, and lesions with low uptake warrant integration with cross-sectional imaging features, tumour kinetics, and clinical context. Importantly, [^99m^Tc]-sestamibi SPECT/CT has minimal utility for systemic staging and should not be used as a replacement for conventional staging investigations. Emerging health economic analyses suggest that the selective use of sestamibi imaging in indeterminate cases may reduce overtreatment and be cost-effective by avoiding unnecessary nephrectomy for benign lesions [20].

### 3.5. Limitations and Pitfalls

Several limitations must be recognised. Biological overlap between oncocytoma, HOCT, and chromophobe RCC can result in false-positive sestamibi uptake, while a minority of oncocytomas with low mitochondrial content may appear sestamibi-negative [15]. Variability in acquisition protocols, uptake thresholds, and reference standards (biopsy versus surgical pathology) contributes to heterogeneity across studies and limits reproducibility between institutions.

**Table 1:** Key studies assessing sestamibi SPECT/CT for differentiating oncocytic from malignant renal lesions. The technique exploits mitochondrial avidity, demonstrating pooled sensitivity and specificity near 90% for benign vs. malignant distinction. Abbreviations: HOCT = hybrid oncocytic/chromophobe tumour; MDR-1 = multidrug resistance protein-1; sens = sensitivity; spec = specificity; PPV = positive predictive value; NPV = negative predictive value. Where confidence intervals were not reported in original studies, point estimates are shown.

Study	Design/Population	Reference Standard	Key Diagnostic Metrics	Histology Focus	Clinical Interpretation
Rowe et al. [14]	Translational + early clinical validation; ~50 renal tumours	Histopathology + molecular correlation	Qualitative uptake correlated with mitochondrial density; inverse correlation with MDR-1 expression	Oncocytoma, HOCT, ccRCC	Established biological mechanism underpinning sestamibi avidity
Gorin et al. [15]	Prospective, single-centre; *n* = 50 cT1 solid renal masses	Surgical histopathology	Sensitivity 87.5% (95% CI 47.4–99.7%); specificity 95.2% (95% CI 83.8–99.4%)	Oncocytoma/HOCT vs. RCC	First prospective clinical validation; strong discrimination in enriched cohort
Sistani et al. [7]	Prospective; *n* = 29 patients (31 masses)	Surgical histopathology	Sensitivity 100%, specificity 96% (CIs not reported)	Oncocytoma/HOCT vs. RCC	Excellent performance in highly enriched indeterminate cohort
Viswambaram et al. [17]	Prospective; *n* = 74 small renal masses (<4 cm)	Histopathology	Sensitivity 89% (95% CI 77–95%); specificity 73% (95% CI 45–91%); AUC 0.883	Mixed benign and malignant	Demonstrated limits of specificity; tumour/kidney ratio cut-off 0.41
Parihar et al. [16]	Retrospective; *n* = 36 solid renal masses	Histopathology	Sensitivity 66.7%; specificity 89.5%; quantitative ratio ≥ 0.46 improved accuracy (87.5% sens/86.7% spec)	Mixed	Quantitative thresholds improve performance but remain cohort-specific
Schober et al. [18]	Retrospective, real-world implementation; *n* = 71 patients (88 masses)	Histopathology or biopsy	~20% of sestamibi-negative lesions benign; NPV ~80% (CIs not reported)	Mixed	Highlights false-negative risk; supports adjunctive rather than definitive use
Basile et al. [4]	PRISMA-registered meta-analysis; 8 studies (501 masses)	Histopathology	Pooled sensitivity 89% (95% CI 70–97%); specificity 89% (95% CI 86–92%); PPV 63%, NPV 98%	Oncocytoma/HOCT vs. RCC	Best pooled evidence supporting sestamibi for benign–malignant risk stratification
Warren et al. [5]	Systematic review of imaging in T1 renal tumours	Various	Qualitative synthesis (no pooled metrics)	Small renal masses	Confirms sestamibi as most validated functional tracer for renal mass characterisation

## 4. ^18^F-Flurodeoxyglucose (FDG) PET/CT Imaging in RCC

### 4.1. Biological Rationale

[^18^F]FDG is the most widely utilised radiotracer in oncologic imaging, reflecting increased glycolytic metabolism within malignant cells including lung, colorectal, breast, and head and neck cancers [21], and is integral to staging and treatment monitoring across many solid tumours. In renal cell carcinoma (RCC), however, its performance is context-dependent.

### 4.2. Role in Localised Disease

For primary renal tumours, [^18^F]FDG-PET demonstrates limited diagnostic accuracy. In a meta-analysis by Wang et al. [22], the pooled sensitivity for primary RCC detection was 62% (95% CI 49–74%), with a specificity of 88% (95% CI 47–100%) and substantial heterogeneity (I^2^ = 82.9%). Physiological urinary [^18^F]FDG excretion obscures intrarenal lesions, and technical measures such as forced diuresis improve visualisation but do not meaningfully enhance diagnostic accuracy [23]. As a result, [^18^F]FDG-PET/CT is not recommended for routine primary renal mass characterisation.

In contrast, [^18^F]FDG-PET/CT performs substantially better in extra-renal disease. Wang et al. reported a pooled sensitivity and specificity of 79% (95% CI 71–86%) and 90% (95% CI 82–95%) on a scan basis, and 84% (95% CI 75–91%) and 91% (95% CI 72–99%) on a lesion basis for metastatic detection [22]. Consistent with this, a subsequent meta-analysis by Ma et al. [24] reported a pooled sensitivity and specificity of 86% (95% CI 88–93%) and 88% (95% CI 84–91%) for the detection of metastatic or recurrent RCC (AUC 0.93), with consistently high performance in bone and lymph node metastases and more variable sensitivity in the liver due to physiological background uptake.

Beyond detection, [^18^F]FDG uptake carries prognostic significance. In prospective single-centre cohorts, a higher SUV_max_ has been associated with inferior outcomes, including an SUV_max_ ≥ 8.8 predicting worse overall survival in advanced RCC [25] and an SUV_max_ > 3.8 associated with shorter progression-free survival in papillary RCC [26]. As these thresholds derive from individual institutional cohorts rather than pooled analyses, they should be interpreted as biologically informative rather than universally generalisable.

Collectively, [^18^F]FDG-PET/CT is best positioned as a complementary tool for risk stratification, prognostication, and treatment monitoring in metastatic or recurrent RCC rather than for primary tumour diagnosis.

### 4.3. Metastatic and Recurrent Disease

[^18^F]FDG-PET/CT demonstrates clearer clinical value in metastatic and recurrent RCC (Table 2). Approximately 10–15% of patients present with metastases at diagnosis, and up to 20% of the remainder develop metastatic disease during follow-up [27].

In a single-centre retrospective study, Bertagna et al. [28] evaluated 68 post-nephrectomy RCC patients undergoing [^18^F]FDG-PET/CT for suspected recurrence or metastases. Using histopathology and clinical follow-up as reference standards, [^18^F]FDG-PET/CT demonstrated a sensitivity of 82%, specificity of 100%, PPV of 100%, NPV of 66.7%, and overall accuracy of 86.8%. Detection rates increased with Fuhrman grade (33% in G2, 47% in G3, and 65% in G4), supporting an association between [^18^F]FDG uptake and tumour aggressiveness, although statistical correlation with grade was not observed.

In a diagnostic accuracy meta-analysis, Ma et al. [24] pooled data from 14 studies comprising 1168 patients evaluated for metastatic or recurrent RCC. FDG-PET/CT demonstrated a pooled sensitivity of 86% (95% CI 88–93%) and specificity of 88% (95% CI 84–91%), with an AUC of 0.93. Performance was strongest for osseous, lymph node, and soft-tissue metastases, while sensitivity was more variable for hepatic lesions due to physiological background uptake. The pooled negative likelihood ratio was 0.18 (95% CI 0.12–0.26), supporting the reliable exclusion of metastatic disease when scans are negative.

Beyond detection, [^18^F]FDG uptake conveys prognostic significance. In prospective single-centre cohorts, an SUV_max_ ≥ 8.8 predicted worse overall survival in advanced RCC [25], while an SUV_max_ > 3.8 was associated with shorter progression-free survival in papillary RCC [26]. These thresholds derive from individual institutional cohorts rather than pooled analyses and should be interpreted as biologically informative rather than universally generalisable.

In therapeutic monitoring, [^18^F]FDG-PET/CT has been explored as an early biomarker of response to tyrosine kinase inhibitors, where size-based CT criteria may be insensitive to biological change. In prospective pilot studies by Lydrel et al. [29], [^18^F]FDG-PET/CT was performed at baseline and early during therapy (approximately 4–6 weeks after initiation) and demonstrated a significant early reduction in mean [^18^F]FDG uptake of approximately 25% after 1–2 months of sorafenib, preceding relatively modest changes in lesion size on CT. Patients with larger early reductions in [^18^F]FDG uptake showed longer overall survival, although early metabolic response did not reliably predict progression-free survival.

Similarly, Vercellino et al. [30] reported that [^18^F]FDG-PET/CT performed after one cycle of sunitinib (day 42) demonstrated metabolic responses largely concordant with later CT-based assessment after two cycles (day 84), suggesting that early SUV_max_ changes may reflect treatment activity before morphologic response is apparent. However, these findings derive from small single-centre cohorts, and metabolic response has not been shown to independently predict progression-free or overall survival beyond established radiographic criteria. Consequently, [^18^F]FDG-PET/CT remains an investigational adjunct for early response assessment rather than a validated monitoring tool in routine RCC care.

**Table 2:** This table summarises key clinical studies assessing the diagnostic and prognostic utility of [^18^F]FDG PET/CT in renal cell carcinoma across primary, recurrent, and metastatic settings. Early studies highlighted limited sensitivity for primary tumours due to physiological urinary excretion, while later meta-analyses demonstrated high accuracy for detecting metastatic or recurrent disease. Quantitative parameters such as SUV_max_ correlate with tumour grade, metabolic aggressiveness, and survival outcomes, underscoring the evolving role of [^18^F]FDG PET/CT as a complementary biomarker for risk stratification, restaging, and treatment monitoring in advanced RCC.

Study	Design/Population	Disease Setting	Key Diagnostic Metrics	SUV_max_/Thresholds	Clinical Relevance
Montravers et al. [31]	Prospective; 22 RCC patients (gamma-camera PET)	Primary + metastatic	Primary detection: 9/13 lesions detected (≈69% sensitivity); metastatic sites well visualised	Not reported	Early evidence supporting [^18^F]FDG for staging/restaging rather than primary diagnosis
Miyakita et al. [32]	Prospective; 19 RCC pre-nephrectomy	Primary	[^18^F]FDG-positive in 31.5% of tumours; no correlation with GLUT-1	Not reported	Demonstrated limited sensitivity for intrarenal disease
Aide et al. [33]	Prospective; 53 RCC vs. CT	Primary + metastatic	Primary: sensitivity 47%, specificity 80%; Metastatic accuracy 94%	Not reported	PET inferior for primary RCC, but detected additional metastases vs. CT
Kamel et al. [23]	Prospective; 32 patients	Primary	Forced diuresis eliminated urinary artefact in 97%; no improvement in diagnostic accuracy	Not reported	Improved visualisation but not diagnostic performance
Kumar et al. [34]	Prospective; 24 patients (28 masses)	Mixed	Malignancy detection 85%; management altered in 30%	SUV_max_: primary 7.9 ± 4.9; metastatic 6.1 ± 3.4	Adjunctive value for staging and lesion characterisation
Takahashi et al. [35]	Retrospective; 92 tumours	Primary	High-grade vs. low-grade ccRCC differentiated	SUV_max_ cut-off 3.0 → 89% sens, 87% spec	[^18^F]FDG uptake correlates with grade; potential risk stratifier
Namura et al. [25]	Prospective; 26 metastatic RCC	Metastatic	Not diagnostic study	SUV_max_ ≥ 8.8 associated with worse OS (*p* = 0.0012)	SUV_max_ an independent prognostic biomarker
Hou et al. [26]	Retrospective; 66 papillary RCC	Metastatic/recurrent	Sensitivity: 81.5% (staging), 100% (restaging)	SUV_max_ cut-off 5.85 → shorter PFS	[^18^F]FDG uptake predicts PFS and recurrence; 16.7% management change
Bertagna et al. [28]	Retrospective; 68 post-nephrectomy RCC	Recurrent/metastatic	Sensitivity 82%, specificity 100%, accuracy 86.8%	PPV 100%, NPV 66.7%	High specificity for restaging; detects occult disease
Wang et al. [22]	Meta-analysis; 14 studies	Primary vs. extrarenal	Primary lesions: pooled sens 62% (95% CI 49–74%), spec 88% (95% CI 47–100%); Extrarenal lesions (PET/CT): sens 91%, spec 88%	Not applicable	Demonstrated markedly better performance for extrarenal disease
Ma et al. [24]	Meta-analysis; 15 studies (*n* = 1168)	Metastatic/recurrent	Pooled sensitivity 86% (95% CI 88–93%), specificity 88% (95% CI 84–91%)	AUC 0.93; DOR 42	Strong evidence supporting [^18^F]FDG PET/CT for restaging
Verhoeff et al. [36]	Prospective; 48 metastatic ccRCC	Metastatic	Not diagnostic accuracy study	High [^18^F]FDG uptake → shorter watchful waiting (9 vs. 36 mo; HR 5.6)	[^18^F]FDG uptake adds prognostic value
Hu et al. [37]	Case series; 6 collecting duct carcinoma	Rare aggressive subtype	All lesions [^18^F]FDG -avid	SUV_max_ > 2.5	[^18^F]FDG useful in aggressive non-ccRCC subtypes

### 4.4. Clinical Perspective

Although [^18^F]FDG-PET/CT is not routinely recommended in EAU (2025) [9], AUA (2021) [10], or NCCN (2025) [12] guidelines for initial diagnosis or staging of RCC, it retains a role in selected clinical contexts. NCCN 2025 guidelines acknowledge its potential utility in advanced or biologically aggressive disease, particularly in stage IV RCC and in fumarate hydratase-deficient or succinate dehydrogenase-deficient subtypes, where [^18^F]FDG uptake may be disproportionately high and clinically informative. Accordingly, [^18^F]FDG-PET/CT is best positioned as a complementary imaging modality for detecting occult metastases, refining prognostic stratification, and monitoring treatment response.

## 5. Prostate-Specific Membrane Antigen (PSMA) PET/CT in RCC

### 5.1. Biological Rationale

PSMA is a transmembrane glycoprotein physiologically expressed in prostate epithelium and pathologically upregulated in prostate cancer. Crucially, PSMA is also expressed in the endothelial cells of tumour-associated neovasculature across multiple solid malignancies, including RCC. Immunohistochemical work by Baccala et al. [38] demonstrated PSMA positivity in 76% of ccRCC and 31% of chromophobe RCCs, with minimal or absent expression in papillary RCC and angiomyolipoma. This endothelial localisation provides the biological basis for PSMA-targeted PET imaging in RCC, reflecting tumour angiogenesis rather than epithelial phenotype.

### 5.2. Localised Disease: Diagnostic and Biological Evaluation

Early prospective data demonstrated that PSMA PET/CT can non-invasively differentiate malignant from benign renal masses through differences in vascular PSMA expression (Table 3). In a pilot study, Golan et al. [39] evaluated dynamic ^68^Ga-PSMA-11 PET/CT in 27 patients with renal masses, finding significantly higher tracer uptake and slower washout kinetics in malignant lesions (median SUV_max_ 9.4 vs. 3.8; *p* = 0.015) compared with benign tumours, correlating closely with histological PSMA expression.

Intra-patient dual-tracer analysis by Tariq et al. [40] demonstrated concordant PSMA and [^18^F]FDG uptake in 55% of metastatic lesions, with discordance favouring PSMA in 27% and [^18^F]FDG in 18%. Rather than reflecting conflicting performance, these patterns underscore the fact that PSMA and [^18^F]FDG interrogate distinct yet complementary dimensions of tumour biology. [^18^F]FDG uptake reflects increased glycolytic metabolism and is most characteristic of dedifferentiated, high-grade, or treatment-resistant tumour phenotypes [21]. Consequently, [^18^F]FDG-positive/PSMA-negative lesions are biologically intuitive markers of aggressive dedifferentiation and may represent disease less likely to respond to angiogenesis-directed or PSMA-targeted therapies [25].

PSMA uptake reflects tumour neovascular density and angiogenic activity, which are hallmarks of clear-cell RCC. Importantly, angiogenic signalling may remain prominent even in tumours harbouring adverse pathological features. Consistent with this, Wang et al. [41] demonstrated superior lesion detection and significantly higher uptake with PSMA PET/CT compared with [^18^F]FDG PET/CT in primary ccRCC (86% vs. 59%; SUV_max_ 15.7 ± 9.0 vs. 5.1 ± 3.4; *p* < 0.001), closely correlating with immunohistochemical PSMA expression. Similarly, in a single-centre ccRCC-only cohort, Chen et al. [42] prospectively evaluated 72 patients with clear-cell RCC undergoing both PSMA PET/CT and [^18^F]FDG PET/CT prior to surgery, with histopathology as the reference standard. PSMA PET/CT outperformed CT and [^18^F]FDG PET/CT for identifying adverse pathological features, including tumour necrosis and sarcomatoid or rhabdoid differentiation. An SUV_max_ threshold of ≥25.3 on PSMA PET/CT was associated with aggressive pathology, achieving an AUC of 0.90 (*p* < 0.001). This threshold was derived from internal receiver operating characteristic analysis and has not been externally validated; therefore, it should be interpreted as a hypothesis-generating, cohort-specific biomarker rather than a universally applicable cut-off. Gasparro et al. [43] further demonstrated that PSMA uptake correlated with immunohistochemical expression and was associated with inferior survival in oligometastatic RCC.

Taken together, these data indicate that PSMA PET/CT is particularly sensitive for detecting angiogenic, high-risk clear-cell disease, whereas [^18^F]FDG PET/CT preferentially identifies metabolically dedifferentiated escape phenotypes.

### 5.3. Metastatic and Systemic Disease Evaluation

In advanced disease, PSMA PET/CT has demonstrated high sensitivity for detecting metastatic lesions and influencing clinical management in selected cohorts. In a single-centre prospective study, Aggarwal et al. [44] evaluated 37 patients with metastatic RCC (predominantly clear-cell histology) and reported that PSMA PET/CT detected 55% more bone lesions than CT and 50% more than [^18^F]FDG PET/CT, although CT remained superior for liver metastases. Quantitative parameters such as total-lesion PSMA (TL-PSMA) and PSMA total volume (PSMA-TV) correlated strongly with survival, suggesting a potential prognostic role, though thresholds were not externally validated.

In a multicentre observational series, Udovicich et al. [45] evaluated 61 patients with metastatic or oligometastatic RCC (89% clear-cell histology) undergoing PSMA PET/CT. PSMA-avid disease was detected in 84% of cases, and imaging findings resulted in a change in management in 49% of patients, with 48% classified as high-impact changes. These changes were primarily driven by the revised assessment of metastatic burden rather than binary upstaging or downstaging. PSMA PET/CT identified additional metastatic lesions not seen on CT in approximately 25% of patients, most commonly leading to a shift from planned metastasis-directed therapy to systemic therapy or surveillance (approximately 23%). Conversely, approximately 10% of patients initially planned for systemic therapy or surveillance underwent metastasis-directed therapy following PSMA PET/CT.

Similarly, in single-study analyses, Wang et al. [41] demonstrated superior metastatic detection with [^68^Ga]Ga-P16-093 PSMA PET/CT compared with [^18^F]FDG PET/CT (95% vs. 64%), while Gasparro et al. [43] reported additional metastatic deposits identified by PSMA PET/CT in 15% of cases, with an SUV_max_ ranging from 11.8 in vascular lesions to 29.8 in hepatic lesions and a significant correlation between tracer uptake and immunohistochemical PSMA expression.

At the pooled level, a meta-analysis by Singhal et al. [46] including 11 studies and approximately 400 lesions reported a pooled sensitivity and specificity of 87% (95% CI 77–95%) and 100% (95% CI 93–100%) for localised disease, and 92% (95% CI 86–96%) and 97% (95% CI 84–100%) for metastatic detection. Diagnostic accuracy was highest in clear-cell RCC (95%) compared with non-clear-cell subtypes (75%), reinforcing PSMA PET/CT’s performance in angiogenic disease while highlighting histology-dependent limitations.

### 5.4. Clinical Perspective

PSMA PET/CT provides complementary diagnostic and biological information in RCC, particularly in clear-cell disease, by delineating tumour burden and angiogenic activity with high lesion-to-background contrast. Across predominantly observational and single-centre studies, PSMA uptake correlates with pathological aggressiveness and may influence clinical decision-making in both primary and metastatic settings. However, limitations include reduced sensitivity for hepatic metastases, variable uptake in non-clear-cell subtypes, and a lack of prospective outcome-driven validation. Multicentre prospective trials are required to determine whether PSMA-guided management translates into improved oncologic outcomes and to define its optimal integration into diagnostic and therapeutic algorithms.

**Table 3:** Summary of key studies evaluating prostate-specific membrane antigen (PSMA) PET/CT in renal cell carcinoma (RCC). Across both localised and metastatic disease, PSMA PET/CT demonstrates high diagnostic accuracy, strong correlation with histopathological angiogenesis and tumour grade, and frequent management impact. Evidence supports its superiority to [^18^F]FDG PET/CT and conventional imaging in lesion detection—particularly in clear-cell histology—though sensitivity for hepatic metastases and non-clear-cell subtypes remains limited.

Study	Design/Population	Tracer	Comparator/Reference Standard	Key Diagnostic Metrics	Clinical/Biological Impact
Baccala et al. [38]	Translational IHC study; 60 renal tumours	PSMA (IHC)	Histopathology	PSMA expression in tumour neovasculature: 76% ccRCC, 31% chromophobe RCC; minimal in papillary RCC and AML	Established biological rationale for PSMA-targeted imaging in RCC
Golan et al. [39]	Prospective; 27 indeterminate renal masses	^68^Ga-PSMA-11	Surgical histopathology	SUV_max_ malignant vs. benign: 9.4 vs. 3.8 (*p* = 0.015); no formal sens/spec reported	Demonstrated correlation between PSMA uptake, neovascular density, and malignancy
Tariq et al. [40]	Prospective dual-tracer pilot; 11 patients	^68^Ga-PSMA-11 vs. [^18^F]FDG	Conventional imaging + histology	Lesion-level concordance 40%; discordant uptake PSMA 20%, [^18^F]FDG 40%; no pooled accuracy metrics	Dual-tracer imaging altered management in 27%; highlighted biological heterogeneity
Wang et al. [41]	Prospective; 42 ccRCC patients	^68^Ga-PSMA-P16-093	[^18^F]FDG PET/CT + histology	Detection rate: PSMA 86% vs. [^18^F]FDG 59%; SUV_max_ 15.7 ± 9.0 vs. 5.1 ± 3.4 (*p* < 0.001)	PSMA uptake correlated with PSMA IHC, stage, and grade
Aggarwal et al. [44]	Prospective; 37 metastatic RCC	^68^Ga-PSMA-11	CECT ± [^18^F]FDG PET/CT	Bone metastases detected: PSMA 312 vs. CT 202 vs. [^18^F]FDG 198 (*p* < 0.001); no sens/spec reported	PSMA-derived tumour volume (PSMA-TV, TL-PSMA) prognostic for OS
Udovicich et al. [45]	Retrospective; 61 metastatic RCC (84% ccRCC)	^68^Ga-PSMA-11	CT ± [^18^F]FDG PET/CT	Patient-level detection rate 88% vs. [^18^F]FDG 75% (*p* = NS); SUV_max_ PSMA 15.2 vs. [^18^F]FDG 8.0 (*p* = 0.02)	Management intent changed in 49%; single-centre observational data
Chen et al. [42]	Prospective; 72 ccRCC with adverse histology	^68^Ga-PSMA-11	Histopathology + CT/[^18^F]FDG	SUV_max_ ≥ 25.3 predicted adverse pathology (AUC 0.90); threshold internally derived, not cross-validated	Improved identification of necrosis, sarcomatoid/rhabdoid differentiation
Gasparro et al. [43]	Retrospective; 26 RCC (22 ccRCC)	^68^Ga-PSMA-HBED-CC	IHC + CT	PET positivity 65%; SUV_max_ correlated with IHC (*p* = 0.01); SUV_max_ ≥ 7.4 associated with worse OS	Suggested prognostic value; hypothesis-generating
Singhal et al. [46]	Systematic review and meta-analysis; 11 studies, >400 patients	PSMA PET/CT	Mixed (histology/imaging/follow-up)	Pooled sensitivity 92% (95% CI ~88–95%), specificity 97% (95% CI ~93–99%); higher accuracy in ccRCC	

## 6. CAIX-Targeted PET Imaging in Renal Cell Carcinoma

### 6.1. Biological Rationale

CAIX is a hypoxia-inducible transmembrane enzyme that catalyses the reversible hydration of carbon dioxide, maintaining pH homeostasis in hypoxic microenvironments [2]. Its expression is tightly controlled by HIF-1α downstream of *VHL* gene inactivation, a defining molecular event in ccRCC [2]. As a result, CAIX is ubiquitously expressed in >90% of ccRCCs, whereas its expression in normal kidney tissue is restricted to collecting ducts, making it an exceptionally specific biomarker for imaging and therapeutic targeting [47].

CAIX has also been associated with favourable clinical outcomes: higher expression correlates with improved overall survival and better responses to systemic therapy, reflecting the underlying tumour biology of less dedifferentiated disease. Incorporating CAIX expression into RCC prognostic nomograms improves predictive accuracy, particularly in metastatic settings [47]. These insights underpin the development of the CAIX-specific monoclonal antibody girentuximab (cG250) and its radiolabelled derivatives for molecular imaging and targeted therapy in RCC.

### 6.2. Diagnosis of the Primary Renal Mass

#### Early Clinical Validation with ^124^I-Girentuximab (REDECT)

The clinical validation of CAIX as an imaging biomarker began with iodine-124-labelled girentuximab (^124^I-girentuximab). In the landmark REDECT (REnal mass Evaluation for DEtection of Clear cell Tumors) trial, Divgi et al. [48] conducted a prospective, multicentre diagnostic accuracy study evaluating 195 patients with renal masses scheduled for surgical resection. All patients underwent both ^124^I-girentuximab PET/CT and contrast-enhanced CT, with histopathology as the reference standard. In this prospective study, PET/CT achieved a mean sensitivity of 86.2% (95% CI 75.3–97.1%) compared with 75.5% (95% CI 62.6–88.4%) for conventional CT (*p* = 0.023). Mean specificity was likewise superior for PET/CT at 85.9% (95% CI 69.4–99.9%) versus 46.8% (95% CI 18.8–74.7%) for CT (*p* = 0.005). Inter-reader agreement was high for PET/CT (κ 0.87–0.92) and exceeded that of CT (κ 0.67–0.76), with strong intrareader reproducibility. ^124^I-girentuximab was well tolerated, with no tracer-related safety concerns identified. However, the long physical half-life of ^124^I (4.2 days), combined with in vivo deiodination, necessitated delayed imaging several days post-injection and contributed to higher whole-body radiation exposure and background activity. These factors limited clinical throughput and patient convenience, constraining widespread adoption despite favourable diagnostic accuracy. Collectively, REDECT provided the first robust clinical evidence that CAIX-targeted PET/CT could non-invasively identify ccRCC with greater accuracy and reproducibility than conventional anatomic imaging in an enriched surgical cohort, while also highlighting practical limitations related to radiochemistry and workflow.

### 6.3. Transition to ^89^Zr-Girentuximab

Subsequent translational work focused on zirconium-89-labelled girentuximab (^89^Zr-girentuximab), which offers improved imaging characteristics for antibody-based PET. Compared with ^124^I, ^89^Zr demonstrates superior tumour retention and image contrast due to intracellular residualisation following antibody internalisation rather than rapid release after lysosomal degradation [49].

In a prospective cohort of 30 patients with indeterminate renal masses or suspected recurrent ccRCC, Hekman et al. [50] demonstrated that all PET-avid resected lesions were confirmed as ccRCC, yielding a positive predictive value of 100%, while PET-negative lesions remained radiologically stable on follow-up. Imaging findings were associated with a change in clinical management in 36% of patients, most commonly through avoidance of repeat biopsy or refinement of surgical planning in the setting of equivocal conventional imaging. Importantly, management changes occurred through the integration of PET findings with clinical, radiologic, and histopathologic data rather than PET results in isolation.

Although ^89^Zr has a shorter half-life than ^124^I (3.3 vs. 4.2 days), antibody-based imaging still requires delayed acquisition, typically 3–5 days post-injection. While image quality and dosimetry are improved relative to ^124^I, these protocols continue to impose logistical constraints, including multi-day scheduling, reduced scanner flexibility, and increased patient burden compared with same-day PET approaches [51].

#### Phase III Validation: ZIRCON

Definitive contemporary validation of CAIX-targeted PET was provided by the phase III ZIRCON trial [51], a prospective, open-label, multicentre study conducted across 36 centres in nine countries. ZIRCON enrolled 300 patients with a single indeterminate renal mass ≤7 cm who underwent ^89^Zr-girentuximab PET/CT prior to partial or radical nephrectomy. A blinded central review by three independent readers demonstrated a mean sensitivity of 85.5% (95% CI 81.5–89.6%) and specificity of 87.0% (95% CI 81.0–93.1%) for detecting ccRCC. Diagnostic performance was consistent across tumour size subgroups, including lesions ≤ 4 cm and ≤3 cm. Mean PPV exceeded 92%, while NPV ranged from 75 to 78%, reflecting the enriched prevalence of ccRCC in surgically managed indeterminate masses rather than intrinsic test limitations. Inter-reader agreement was excellent (κ > 0.9). Although ZIRCON was a diagnostic rather than interventional study, systematic safety monitoring was appropriate given the intravenous administration of a radiolabelled monoclonal antibody. Tracer-related adverse events were infrequent, predominantly mild, and did not differ meaningfully from background perioperative morbidity, with most serious adverse events occurring post-surgery and deemed unrelated to the imaging agent.

### 6.4. Clinical Role, Workflow Implications, and Limitations

Collectively, CAIX-targeted PET/CT represents the most rigorously validated molecular imaging approach for the non-invasive identification of ccRCC to date. Its principal clinical value lies in the characterisation of indeterminate renal masses, particularly when biopsy is inconclusive, impractical, or carries disproportionate risk, and in refining surgical decision-making by improving diagnostic confidence. Importantly, CAIX PET/CT does not replace histopathology, but serves as a biologically specific adjunct to conventional imaging.

Nevertheless, important limitations remain. Antibody-based tracers require delayed imaging several days post-injection and incur higher cumulative radiation exposure than small-molecule or peptide tracers. These factors constrain clinical throughput, increase scheduling complexity, and reduce patient convenience [51]. Moreover, CAIX expression is largely confined to clear-cell histology, limiting applicability to non-clear-cell RCC subtypes. Negative predictive value is influenced by disease prevalence and cohort enrichment, underscoring the need for careful patient selection and integration with clinical context.

### 6.5. Emergence of Peptide and Small-Molecule CAIX Tracers

To address the logistical and dosimetric limitations of antibody-based CAIX imaging, recent efforts have focused on radiolabelled peptides and small-molecule CAIX inhibitors with rapid pharmacokinetics and same-day imaging feasibility.

[^68^Ga]Ga-DPI-4452 (Debio-0328) is a cyclic CAIX-targeted peptide designed for fast tumour uptake and rapid background clearance. In a first-in-human proof-of-concept study involving three patients with advanced ccRCC, Hofman et al. [52] reported intense tracer uptake (SUV_max_ > 100) with excellent tumour-to-background contrast and clear delineation of metastatic lesions, including sites not visualised on CT or [^18^F]FDG-PET/CT. Renal cortical activity was minimal and no tracer-related safety concerns were observed. However, given the very small sample size and exploratory nature of this study, no conclusions regarding diagnostic accuracy, comparative performance, or clinical impact can be drawn.

From a practical standpoint, generator-based ^68^Ga production enables on-site radiolabelling and single-visit imaging, with optimal acquisition approximately 60 min post-injection. This represents a substantial improvement in workflow efficiency, scanner utilisation, and patient convenience compared with antibody-based approaches. Although DPI-4452 can be labelled with therapeutic radionuclides such as ^177^Lu or ^225^Ac, current evidence supporting CAIX-directed theranostics remains preclinical or early-phase, and such applications should be considered investigational. Ongoing prospective evaluation (NCT05706129) will be required to define feasibility, safety, and therapeutic index.

### 6.6. Staging and Detection of Locoregional or Metastatic Disease

Beyond primary tumour characterisation, CAIX PET/CT may contribute to whole-body disease mapping in metastatic ccRCC, although evidence is currently limited to single prospective cohorts rather than pooled analyses (Table 4). In the IMPACT-RCC study, Verhoeff et al. [53] prospectively evaluated 42 patients with newly diagnosed good- or intermediate-risk metastatic ccRCC using [^89^Zr]Zr-girentuximab PET/CT, [^18^F]FDG-PET/CT, and conventional CT. Across 449 lesions, CAIX PET detected 70% of metastatic sites, compared with 56% for CT and 59% for [^18^F]FDG-PET/CT. When combined with CT, CAIX PET achieved the highest overall detection rate (91%), with the greatest incremental benefit observed for osseous metastases, which are frequently occult on conventional imaging.

Importantly, this was a single-centre prospective study, and no pooled sensitivity or specificity estimates are currently available for CAIX PET/CT in metastatic disease. The quantitative CAIX uptake parameters did not correlate with survival outcomes, reinforcing the fact that established clinical risk models such as the IMDC score remain central to prognostication. Nevertheless, CAIX PET/CT may enhance baseline lesion mapping in selected complex cases and in clinical trial settings where comprehensive disease delineation is required.

**Table 4:** Summary of major studies evaluating antibody- and peptide-based CAIX PET/CT for clear-cell RCC detection and staging. ^89^Zr-girentuximab shows high accuracy for subtype identification, while emerging ^68^Ga-labelled peptides enable rapid, same-day imaging. Abbreviations: CAIX = carbonic anhydrase IX; ccRCC = clear-cell renal cell carcinoma; PET = positron emission tomography; PPV = positive predictive value; NPV = negative predictive value. Where confidence intervals were not reported in original publications, point estimates or qualitative outcomes are shown.

Study	Tracer/Modality	Design/Population	Reference Standard	Key Diagnostic Metrics	Clinical Interpretation
Divgi et al. [48] (REDECT)	^124^I-girentuximab PET/CT	Prospective multicentre diagnostic study; *n* = 195 renal masses pre-resection	Surgical histopathology	Sensitivity 86.2% (95% CI 75.3–97.1%); specificity 85.9% (95% CI 69.4–99.9%); superior to CT (sens 75.5%, spec 46.8%)	First robust clinical validation of CAIX-targeted PET for ccRCC
Cheal et al. [49]	^124^I- vs. ^89^Zr-girentuximab	Preclinical ccRCC xenograft comparison	Ex vivo tumour analysis	^89^Zr demonstrated superior tumour retention and tumour-to-background contrast	Established ^89^Zr as preferred isotope for antibody-based CAIX PET
Hekman et al. [50]	^89^Zr-girentuximab PET/CT	Prospective single-centre; *n* = 30 indeterminate renal lesions or suspected recurrence	Histopathology and imaging follow-up	PPV 100% for PET-avid lesions; sensitivity/specificity not powered; management change in 36%	Supports problem-solving role in indeterminate renal masses
Verhoeff et al. [36] (IMPACT-RCC)	^89^Zr-girentuximab PET/CT vs. [^18^F]FDG PET/CT	Prospective single-centre; *n* = 42 metastatic ccRCC	Composite imaging and follow-up	Lesion detection: CAIX PET 70% vs. CT 56% vs. [^18^F]FDG 59%; highest combined detection with PET + CT (91%)	Improved baseline lesion mapping, particularly for osseous disease
Shuch et al. [51] (ZIRCON)	^89^Zr-DFO-girentuximab PET/CT	Phase III multicentre diagnostic study; *n* = 300 renal masses ≤7 cm	Surgical histopathology	Sensitivity 85.5% (95% CI 81.5–89.6%); specificity 87.0% (95% CI 81.0–93.1%); PPV > 92%; NPV 75–78%	Definitive contemporary validation of CAIX PET for ccRCC identification
Nakaigawa et al. [54] (ZIRDAC-JP)	^89^Zr-DFO-girentuximab PET/CT	Phase I Japanese cohort	Surgical histopathology	Diagnostic performance and biodistribution consistent with ZIRCON (CIs not reported)	Demonstrated cross-population reproducibility
Hofman et al. [52]	^68^Ga-DPI-4452 (CAIX peptide PET/CT)	First-in-human proof-of-concept; *n* = 3 metastatic ccRCC	CT, [^18^F]FDG PET/CT, clinical follow-up	Very high uptake (SUV_max_ > 100); additional lesions detected vs. CT/[^18^F]FDG; no accuracy metrics	Early-phase feasibility only; diagnostic and theranostic roles remain investigational

## 7. Fibroblast Activation Protein Inhibitor (FAPI) PET/CT

### 7.1. Biological Rationale

Fibroblast activation protein (FAP) is a type II transmembrane serine protease expressed predominantly by cancer-associated fibroblasts (CAFs), a key stromal cell population implicated in tumour progression, angiogenesis, immune evasion, and extracellular matrix remodelling [55]. Elevated FAP expression has been associated with poor prognosis and aggressive tumour behaviour across multiple solid malignancies, including breast, pancreatic, and colorectal cancers [55]. In RCC, FAP expression appears enriched in advanced, dedifferentiated, and sarcomatoid phenotypes, reflecting the activation of the tumour stroma rather than tumour-cell-intrinsic biology.

The development of gallium-68-labelled fibroblast activation protein inhibitors (^68^Ga-FAPI) enables the non-invasive visualisation of the tumour microenvironment rather than tumour cells themselves. These tracers demonstrate rapid lesion uptake and low physiological background activity, particularly in the liver, pancreas, and kidneys, allowing high-contrast imaging of stromal-rich disease [55]. While the DOTA-based chelator permits labelling with therapeutic radionuclides such as lutetium-177 or yttrium-90, current clinical evidence primarily supports a diagnostic and biological phenotyping role, and any theranostic application remains investigational.

### 7.2. Clinical Evidence in RCC

Clinical data supporting FAPI PET/CT in RCC remain limited to small single-centre cohorts, with no pooled diagnostic accuracy estimates currently available. In a prospective exploratory study of 20 patients, Civan et al. [56] demonstrated that [^68^Ga]Ga-FAPI-04 PET/CT achieved significantly higher tumour-to-background ratios than [^18^F]FDG PET/CT (median 5.6 vs. 2.1, *p* < 0.001), with higher SUV_max_ observed in lung metastases and locally recurrent lesions. Notably, this study was not designed to estimate sensitivity or specificity, but rather to compare relative contrast and lesion conspicuity between tracers. FAPI uptake was higher in advanced-stage and recurrent disease, suggesting an association with aggressive tumour biology and stromal activation.

High FAPI uptake has also been reported in sarcomatoid RCC metastases in small series, consistent with the established link between fibroblast activation, dedifferentiation, and tumour invasiveness. These observations parallel prior [^18^F]FDG-PET findings in which an elevated SUV_max_ has been associated with poor prognosis and reduced survival in advanced RCC [25]. However, it is important to note that FAPI and [^18^F]FDG interrogate distinct biological processes, namely, stromal activation versus tumour glycolysis, and their uptake patterns should not be interpreted interchangeably.

### 7.3. Comparative and Translational Insights

Early comparative studies suggest complementary rather than competitive roles for FAPI and [^18^F]FDG imaging. FAPI PET/CT appears to offer superior lesion-to-background contrast in soft-tissue, pulmonary, and locoregional disease, particularly in lesions with prominent desmoplastic or stromal components, whereas [^18^F]FDG remains informative for assessing metabolic aggressiveness and treatment response. Oldan et al. [57] highlighted potential parity or superiority of FAPI over [^18^F]FDG in tumours characterised by strong stromal activation, although these conclusions were derived from small heterogeneous cohorts and should be interpreted cautiously.

From a translational perspective, FAPI PET/CT may provide value for biological phenotyping and research stratification rather than definitive diagnostic decision-making. At present, no validated SUV thresholds, sensitivity/specificity estimates, or outcome-driven cut-offs exist for RCC.

### 7.4. Limitations

A major limitation of FAPI imaging is its lack of cancer specificity. In the first systematic review of benign FAPI-avid findings, Bentestuen et al. [58] reported over 2300 benign lesions, most commonly related to vascular remodelling, degenerative joint disease, infection, fibrosis, and wound healing. The available evidence is largely descriptive, dominated by case reports and small cohorts, with no validated SUV cut-offs or pooled diagnostic accuracy estimates. Within the kidney, benign entities such as lipid-poor angiomyolipoma and inflammatory conditions including xanthogranulomatous pyelonephritis may demonstrate intense FAPI uptake and mimic RCC. Diffuse renal uptake has also been associated with renal fibrosis and reduced renal function, further limiting specificity.

Accordingly, FAPI PET/CT requires cautious interpretation and should always be correlated with clinical context and CT or MRI morphology, with particular attention to benign inflammatory, fibrotic, or post-treatment mimics. Although early RCC studies report high lesion conspicuity, robust head-to-head comparative data versus [^18^F]FDG, PSMA, or CAIX imaging are lacking. Consequently, FAPI PET/CT should currently be considered an investigational adjunct rather than a standalone diagnostic modality.

### 7.5. Current Role and Future Directions

At present, FAPI PET/CT remains investigational in RCC, with its principal value lying in biological characterisation and hypothesis-generating research. Future prospective studies are required to establish reproducible interpretation criteria, define incremental benefit over established tracers, and determine whether FAPI-guided strategies translate into meaningful clinical impact. Until such evidence emerges, FAPI-based theranostic applications should be restricted to early-phase clinical trials.

## 8. Dual-Tracer PET/CT Imaging in RCC

The heterogeneous metabolic and molecular landscape of RCC poses inherent limitations for single-tracer imaging. [^18^F]FDG, PSMA, and CAIX interrogate distinct biological processes—glycolytic metabolism, tumour neovascularisation, and hypoxia-driven CAIX expression (Table 5), respectively—and dual-tracer approaches have therefore been explored to provide a more comprehensive, intra-patient assessment of tumour biology. By integrating complementary metabolic and molecular information, dual-tracer PET/CT may better characterise inter- and intra-lesional heterogeneity across disease sites.

Evidence supporting dual-tracer imaging in RCC is currently limited to small exploratory cohorts, and reported performance metrics derive from single-centre or subgroup analyses rather than pooled estimates. In an intra-individual comparison by Tariq et al. [40] eleven patients with localised, recurrent, or metastatic RCC underwent both [^18^F]FDG and PSMA PET/CT. Concordant tracer uptake was observed in approximately 55% of assessed lesions, while discordant uptake was seen in the remainder, favouring PSMA in 27% and [^18^F]FDG in 18% of lesions. These patterns were interpreted as reflecting differential angiogenic and metabolic phenotypes rather than discordant diagnostic performance. Dual-tracer imaging was equivalent or superior to contrast-enhanced CT in selected cases and resulted in a change in clinical management in 27% of patients, including the refinement of surgical planning and targeted treatment of metastatic lesions. Importantly, all management decisions were made in conjunction with conventional imaging and histopathologic confirmation rather than PET findings alone.

Complementary findings were reported by Udovicich et al. [45] in a subgroup analysis of 40 patients who underwent both PSMA and [^18^F]FDG PET/CT within a larger PSMA PET cohort. At the patient level, PSMA PET/CT demonstrated a higher detection rate than [^18^F]FDG PET/CT (88% vs. 75%), although this difference did not reach statistical significance. Concordant PSMA-positive/[^18^F]FDG-positive disease predominated, while discordant uptake patterns were less frequent and most commonly PSMA-positive/[^18^F]FDG-negative. Among patients with concordantly positive disease, tracer uptake intensity was significantly higher on PSMA PET/CT than [^18^F]FDG PET/CT (SUV_max_ 15.2 vs. 8.0; *p* = 0.02), reflecting stronger angiogenic signal intensity. These data were derived from a single cohort and were not powered to establish sensitivity, specificity, or predictive values.

Biologically, discordant uptake patterns likely reflect underlying tumour heterogeneity. [^18^F]FDG-positive/PSMA-negative lesions are consistent with dedifferentiated, glycolytically active, or treatment-resistant tumour phenotypes, whereas PSMA-positive/[^18^F]FDG-negative lesions may retain angiogenic signalling typical of clear-cell RCC. Dual-tracer imaging may therefore assist in lesion phenotyping and the identification of heterogeneous or oligometastatic disease in selected clinical or research settings, particularly when considering metastasis-directed therapy or enrolment in biomarker-driven trials. However, such applications remain hypothesis-generating.

### 8.1. Limitations and Pragmatic Considerations of Dual-Tracer Imaging

Despite its biological appeal, the routine clinical adoption of dual-tracer PET imaging is constrained by practical and economic limitations. Dual-tracer protocols typically require multiple imaging sessions, increasing patient burden, logistical complexity, and cumulative radiation exposure. Even when same-day imaging is feasible, tracer availability, scheduling, and scanner throughput present challenges in high-volume centres.

From a health economic perspective, the incremental cost of dual-tracer imaging is substantial, encompassing radiotracer production, scanner time, and specialist reporting. In contrast, decision-analytic models evaluating the selective use of [^99m^Tc]-sestamibi SPECT/CT in indeterminate renal masses have shown cost-effectiveness when imaging avoids unnecessary surgery for benign lesions, with modelling suggesting that preventing nephrectomy in approximately 10–20% of cases offsets the additional imaging cost [20]. In the absence of prospective evidence demonstrating improved patient-level outcomes—such as survival benefit, reduced toxicity, or avoidance of ineffective therapy—widespread reimbursement is difficult to justify. Accordingly, management impact, negative predictive value, and the exclusion of futile treatment may be more clinically meaningful endpoints than sensitivity alone when evaluating the role of dual-tracer imaging.

Overall, current evidence supports dual-tracer PET/CT as a selective, problem-solving or research tool rather than a generalisable standard of care. Prospective multicentre studies with standardised acquisition protocols, histopathologic correlation, and outcome-based endpoints are required to define its incremental value over single-tracer imaging and conventional modalities.

**Table 5:** Summary of PET radiotracers evaluated in renal cell carcinoma (RCC), outlining their molecular targets, diagnostic performance, prognostic insights, and key limitations across clinical applications including diagnosis, staging, and therapy response assessment.

Tracer	Molecular Target/Mechanism	Key Diagnostic Performance	Prognostic/Functional Insights	Advantages	Limitations
**[^18^F]FDG**	Glucose metabolism via GLUT-1 and hexokinase pathways	Primary RCC: pooled sensitivity 62%, specificity 88% (Wang et al. [24]); metastatic/recurrent disease: pooled sensitivity 86%, specificity 88% (Ma et al. [24])	High SUV_max_ associated with aggressive biology: SUV_max_ ≥ 8.8 predicts worse OS (Namura et al. [26]); SUV_max_ > 3 associated with high-grade ccRCC (Takahashi et al. [35]); SUV_max_ > 5.85 predicts shorter PFS in papillary RCC (Hou et al. [59])	Widely available; reflects tumour metabolism; prognostic biomarker; useful for therapy response monitoring	High renal/urinary background; limited sensitivity for small or low-grade primary tumours
**[^68^Ga]Ga-PSMA-11/[^18^F]DCFPyL**	PSMA expression in tumour neovasculature	Metastatic RCC: sensitivity ~84–91%, specificity ~88% (single-centre and multicentre cohorts) (Udovicich et al.) [45]	Higher SUV_max_ correlates with angiogenic activity and aggressive pathology; complementary to [^18^F]FDG for intra-patient heterogeneity	Excellent lesion-to-background contrast; detects occult disease missed by CT or [^18^F]FDG; management impact in ~40–50% of cases	Reduced uptake in non-ccRCC; physiological renal and salivary uptake; availability and regulatory access
**[^89^Zr]Zr-DFO-girentuximab/[^124^I]girentuximab**	CAIX overexpression driven by VHL–HIF pathway in ccRCC	Primary ccRCC: sensitivity ~85–90%, specificity ~80–90% in prospective trials (Divgi et al. [48]; Shuch et al. [51])	Uptake reflects CAIX expression and angiogenic phenotype; reduced uptake may indicate dedifferentiation (single-centre data)	Highly specific for ccRCC; low non-renal background; robust prospective validation	Slow antibody kinetics (multi-day imaging); higher radiation exposure; limited utility in non-ccRCC
**[^99m^Tc]-sestamibi SPECT/CT**	Mitochondrial membrane potential (oncocytic tumours)	Differentiation of oncocytoma/HOCT vs. RCC: pooled sensitivity ~89%, specificity ~89% (Basile et al. [4]; Warren et al. [5])	Supports benign risk stratification; may reduce unnecessary surgery	Distinguishes oncocytic tumours; widely available SPECT platform; cost-effective	Limited role in staging; overlap with chromophobe RCC; modest NPV
**[^68^Ga]Ga-FAPI**	Fibroblast activation protein (tumour stroma)	Early single-centre studies show high uptake in RCC metastases; no pooled accuracy estimates	May reflect tumour microenvironment and stromal activation	High tumour-to-background contrast; minimal urinary excretion	Limited clinical validation; non-specific uptake in benign inflammatory/fibrotic conditions
**Dual-tracer imaging ([^18^F]FDG ± PSMA or CAIX)**	Combined metabolic and angiogenic/CAIX targeting	Improved detection of heterogeneous disease; upstaging or management change in ~15–30% (exploratory cohorts (Udovicich et al. [45])	Identifies dedifferentiated phenotypes (FDG+/PSMA−); supports therapy selection and trial enrolment	Captures intra-patient heterogeneity; clarifies equivocal single-tracer findings	Requires multiple scans; higher cost and radiation; incremental benefit over single-tracer imaging unproven

### 8.2. Key Pitfalls and Interpretative Caveats in PET and SPECT Imaging for RCC

#### 8.2.1. [^18^F]FDG PET/CT

- Primary tumours: Limited sensitivity for intrarenal lesions due to urinary tracer excretion and heterogeneous glycolytic activity, particularly in low-grade and organ-confined disease.
- Biological variability: Low [^18^F]FDG uptake does not exclude malignancy; uptake correlates more strongly with grade and dedifferentiation than with tumour presence alone.
- Strengths remain in metastatic and restaging settings, particularly for bone, nodal, and soft-tissue disease.

#### 8.2.2. PSMA PET/CT

- Organ-specific limitations: Reduced sensitivity for liver metastases due to physiological hepatic uptake; caution required when excluding hepatic disease.
- Histological dependence: Highest performance in clear-cell RCC; uptake is lower and more variable in non-clear-cell subtypes.
- Interpretation caveat: PSMA reflects tumour neovasculature rather than tumour cells themselves and should not be interpreted as a prostate-cancer-specific signal.

#### 8.2.3. CAIX-Targeted PET/CT (^124^I- or ^89^Zr-Girentuximab)

- Subtype specificity: Largely restricted to clear-cell RCC; limited utility in non-clear-cell histologies.
- Logistical constraints: Antibody-based tracers require delayed imaging several days post-injection and are associated with higher radiation exposure, limiting throughput and convenience.
- Negative predictive value: Influenced by cohort enrichment and disease prevalence; a negative scan does not exclude malignancy.

#### 8.2.4. [^99m^Tc]-Sestamibi SPECT/CT

- False positives: Uptake may occur in chromophobe RCC due to shared mitochondrial biology.
- False negatives: A subset of oncocytomas with low mitochondrial density may be sestamibi-negative.
- Scope: Limited to primary renal mass characterisation; not suitable for staging or metastatic assessment.

#### 8.2.5. FAPI PET/CT

- Lack of specificity: Uptake occurs in numerous benign inflammatory, fibrotic, and reparative processes, including lipid-poor angiomyolipoma and xanthogranulomatous pyelonephritis.
- Evidence base: RCC data remain preliminary, with small cohorts and limited histopathological validation.
- Clinical role: Best regarded as investigational and interpreted only with careful correlation to CT/MRI and clinical context.

## 9. Conclusions

Molecular imaging in RCC has progressed beyond anatomical delineation to functional characterisation of tumour biology. While [^18^F]FDG remains the most accessible tracer, its limitations in primary RCC highlight the need for more specific agents. PSMA- and CAIX-targeted PET/CT have shown promise in capturing neovasculature and hypoxia-related expression patterns, while [^68^Ga]Ga-FAPI expands the imaging spectrum to the tumour microenvironment. Each tracer offers unique biological insight, whether metabolic, angiogenic, or stromal, reflecting the inherent heterogeneity of RCC.

Emerging dual-tracer approaches combining [^18^F]FDG with PSMA or CAIX further enhance lesion characterisation, aiding distinction between indolent and aggressive disease and refining selection for metastasis-directed or systemic therapies. As prospective multicentre studies mature, standardised interpretation, harmonised reporting frameworks, and integration with molecular pathology will be crucial to translate these tools into clinical decision-making. Ultimately, PET-based molecular imaging represents a powerful adjunct in the precision management of RCC, shifting from detection alone towards a biologically informed paradigm that guides prognosis, treatment selection, and real-time therapeutic monitoring.

## 10. Take-Home Messages

1. [^18^F]FDG-PET/CT provides prognostic value in RCC by correlating metabolic activity with tumour grade and survival.
2. Sestamibi SPECT/CT accurately differentiates benign oncocytic tumours from malignant RCC, reducing unnecessary surgery.
3. PSMA-PET/CT outperforms [^18^F]FDG in clear-cell RCC, altering management in up to half of metastatic cases.
4. CAIX and FAPI tracers expand PET imaging into hypoxia and stromal biology, enabling theranostic precision.

## Data Availability

No new data were created or analyzed in this study. Data sharing is not applicable to this article.
